# Supplementary material for: Hollow-core optical fibre sensors for operando Raman spectroscopy investigation of Li-ion battery liquid electrolytes
Source: Nat Commun. 2022 Mar 28;13:1651. doi: 10.1038/s41467-022-29330-4 (PMC8960792; doi:10.1038/s41467-022-29330-4)
Supplement: Supplementary file 1 — Supplementary Information [file 41467_2022_29330_MOESM1_ESM.pdf]

Supplementary Information

Hollow-core optical fibre sensors for operando Raman spectroscopy investigation of Li-ion battery liquid electrolytes

Ermanno Miele,[1,2,3] Wesley M. Dose,[2,3,4] Ilya Manyakin,[1] Michael H. Frosz,[5] Zachary Ruff,[2,3] Michael F.L. De Volder,[4,3] Clare P. Grey,[2,3*] Jeremy J. Baumberg,[1,3*] and Tijmen G. Euser[1,3*]

[1] Nanophotonics Centre, Department of Physics, Cavendish Laboratory, University of Cambridge, CB3 0HE, Cambridge, United Kingdom

[2] Department of Chemistry, University of Cambridge, Lensfield Road, CB2 1EW, Cambridge, UK

[3] The Faraday Institution, Quad One, Harwell Science and Innovation Campus, Didcot OX11 0RA, Oxford, UK

[4] Institute for Manufacturing, Department of Engineering, University of Cambridge, 17 Charles Babbage Road, CB3 0FS, Cambridge, UK

[5] Max Planck Institute for the Science of Light, Staudtstr. 2, 91058, Erlangen, Germany

*e-mail: te287@cam.ac.uk, jjb12@cam.ac.uk, cpg27@cam.ac.uk

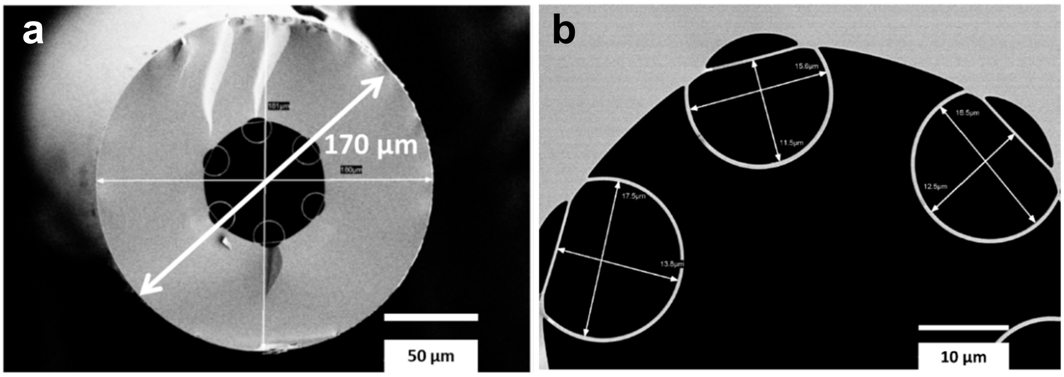

Supplementary Figure 1 | SEM Images of hollow-core fibre. *(**a**) Fibre comprises a single ring negative curvature fibre with an outer diameter of 174 µm and an internal hollow-core diameter measuring 36 µm, with (**b**) 6 inner capillaries of 16 µm in diameter. The glass capillaries are made from Heraeus Suprasil 300 silica glass.*

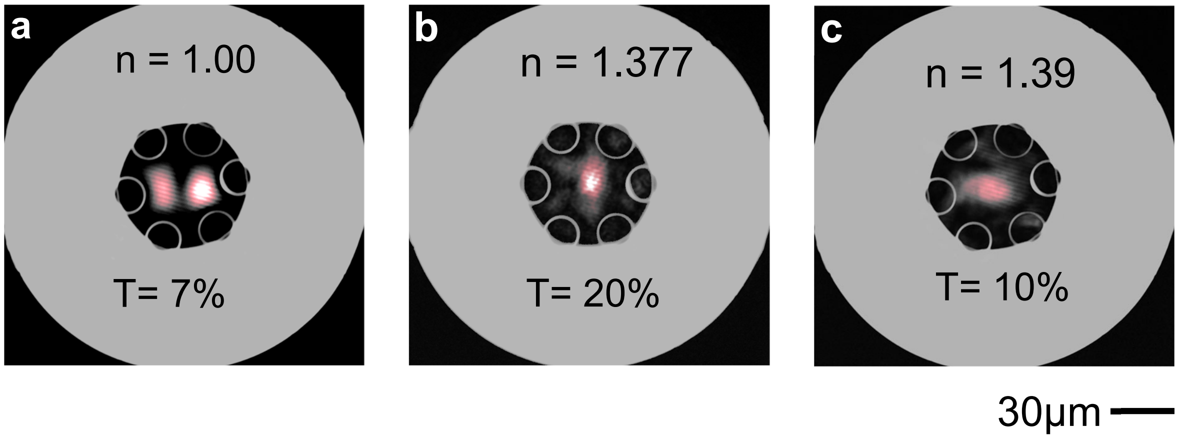

Supplementary Figure 2 | Fibre transmission properties at 785 nm. *Transmitted optical modes at 785 nm for three filling materials with different refractive indices: (**a**) air, (**b**) IPA, and (**c**) EC:EMC (3:7). The CCD mode images are overlaid with optical microscope images taken in the same setup, using a white-light source to illuminate the glass cladding structure. The transmitted power was measured using a Si photodiode power meter with typical values between 10% and 20% for refractive indices between 1.37 and 1.42.*

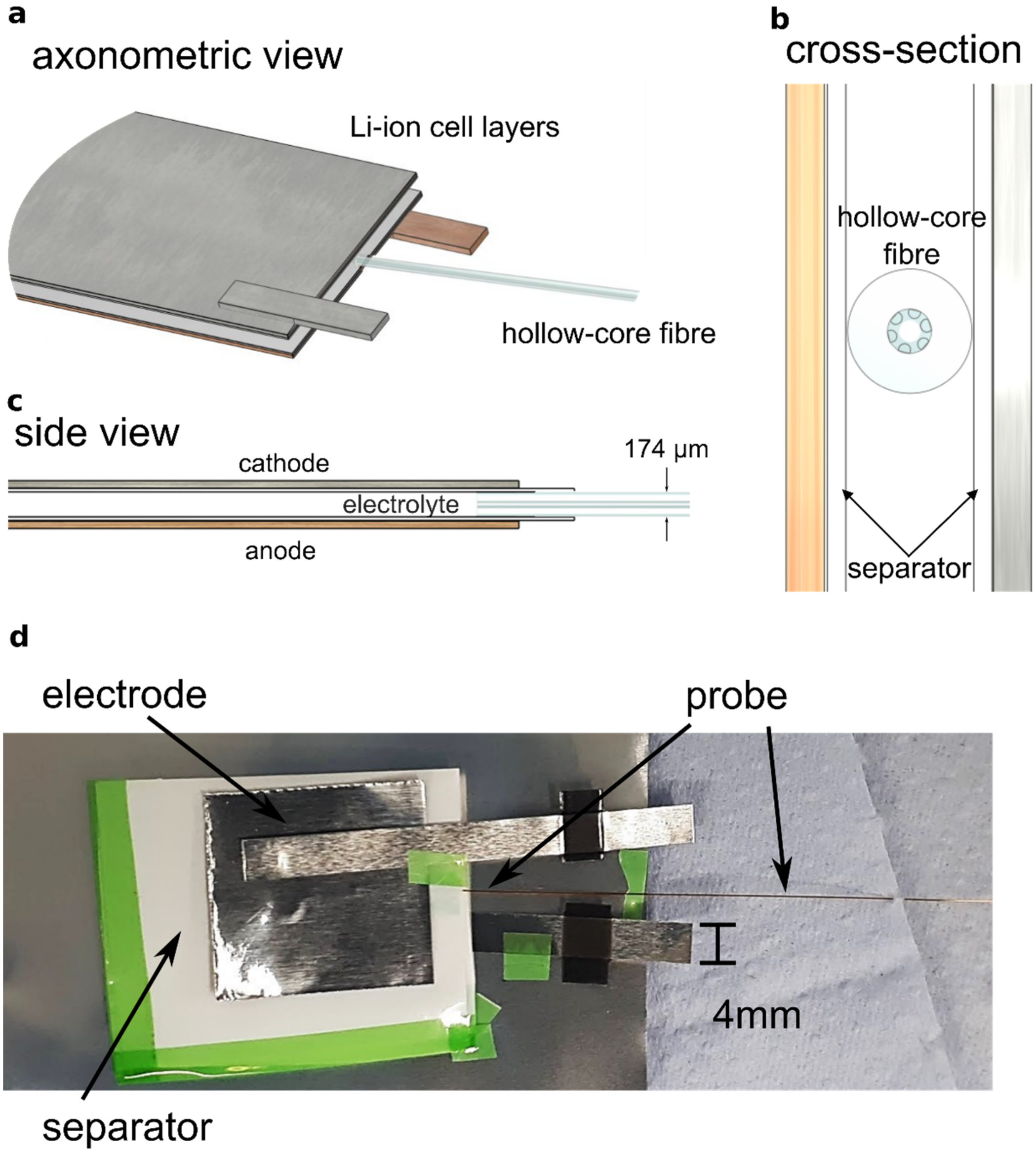

Supplementary Figure 3 |Li-ion cell stack geometry and assembly. *(**a**-**b**) The hollow-core fibre is embedded in the electrolyte compartment, protected by two 25 µm-thick layers of monolayer PE polymer separator (MTI). (**c**) Cross-section of the hollow-core fibre position within the cell. The outer diameter of the fibre is 174 µm. (**d**) Photograph of cell assembly using square electrodes (as used for the multi-cycle experiment in **Figure 6**). An identical assembly method (but with circular electrodes) was used for the data in **Figures 3-5**.*

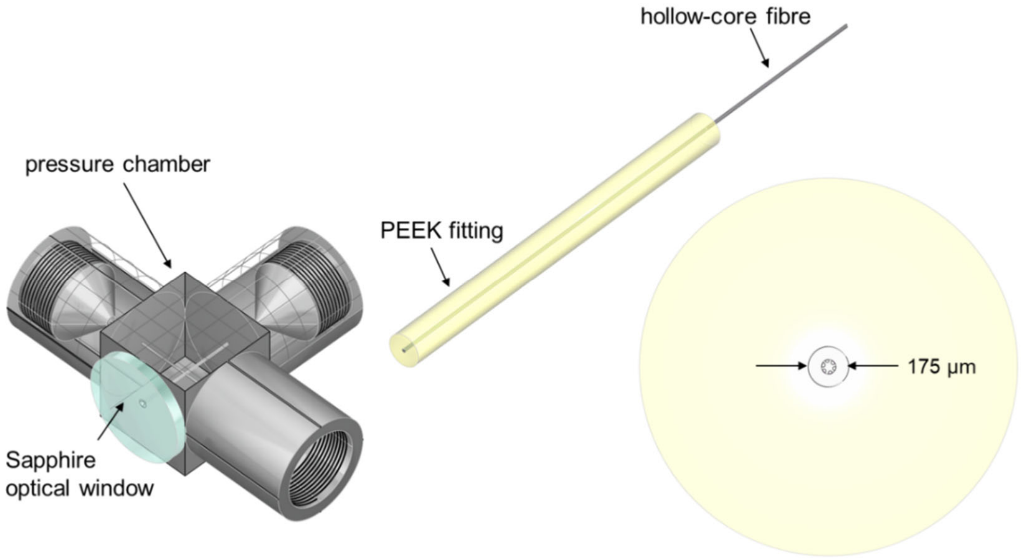

***Supplementary Figure 4 | Low dead-volume pressure chamber for light coupling and microfluidic actuation**. An HPLC (High Performance Liquid Chromatography) tee-connector (Idex UH-422) is customized to connect the fibre to the syringe pump and allow optical access. A 1 mm thick sapphire optical access window (Edmund optics #43-366) is mounted to the cell using an optical adhesive. The fibre is connected to an access port by embedding it in a 1/16" outer diameter, 229 µm inner diameter PEEK sleeve (Upchurch F-227) and fixed with a finger-tight fitting (Upchurch F-120, not shown).*

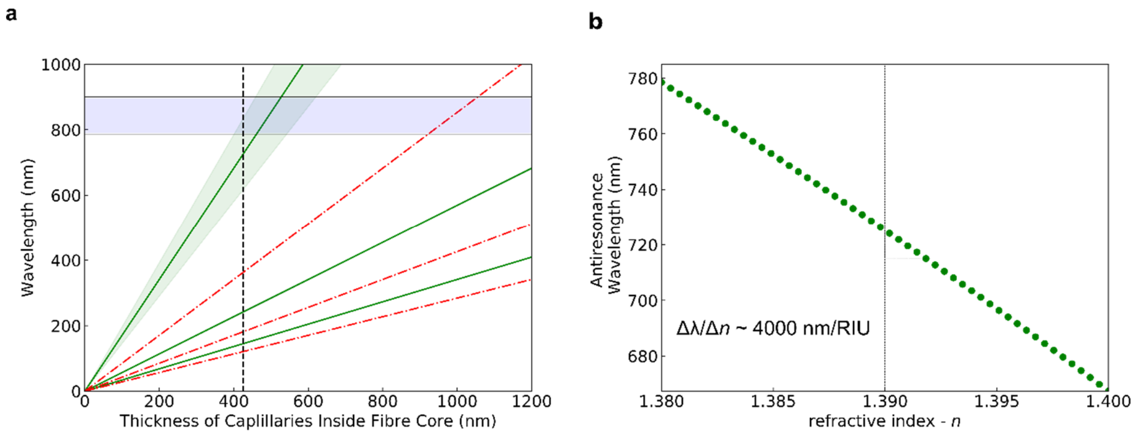

***Supplementary Figure 5 | Anti-resonance and resonance spectral position.** (**a**) Position of the anti-resonances (green lines) and resonances (red lines) of the fibre, as predicted by the ARROW model[1] for n_{core}= 1.39 and n_{glass}= 1.454. The shaded band indicates a ~30% spectral width of the first guidance band that is typical for ARROW waveguides.[1] The vertical dashed line indicates the average wall thickness of the capillaries in the fibre core (425 nm), the horizontal shaded band indicates the spectral region of interest (785 – 910 nm). The Raman band lies well above the first loss resonance (λ= 380 nm), and therefore reasonable guidance is expected. (**b**) Dependence of the anti-resonance position on liquid-core refractive index. The anti-resonance position shifts by -4000* nm *per refractive index unit (RIU).*

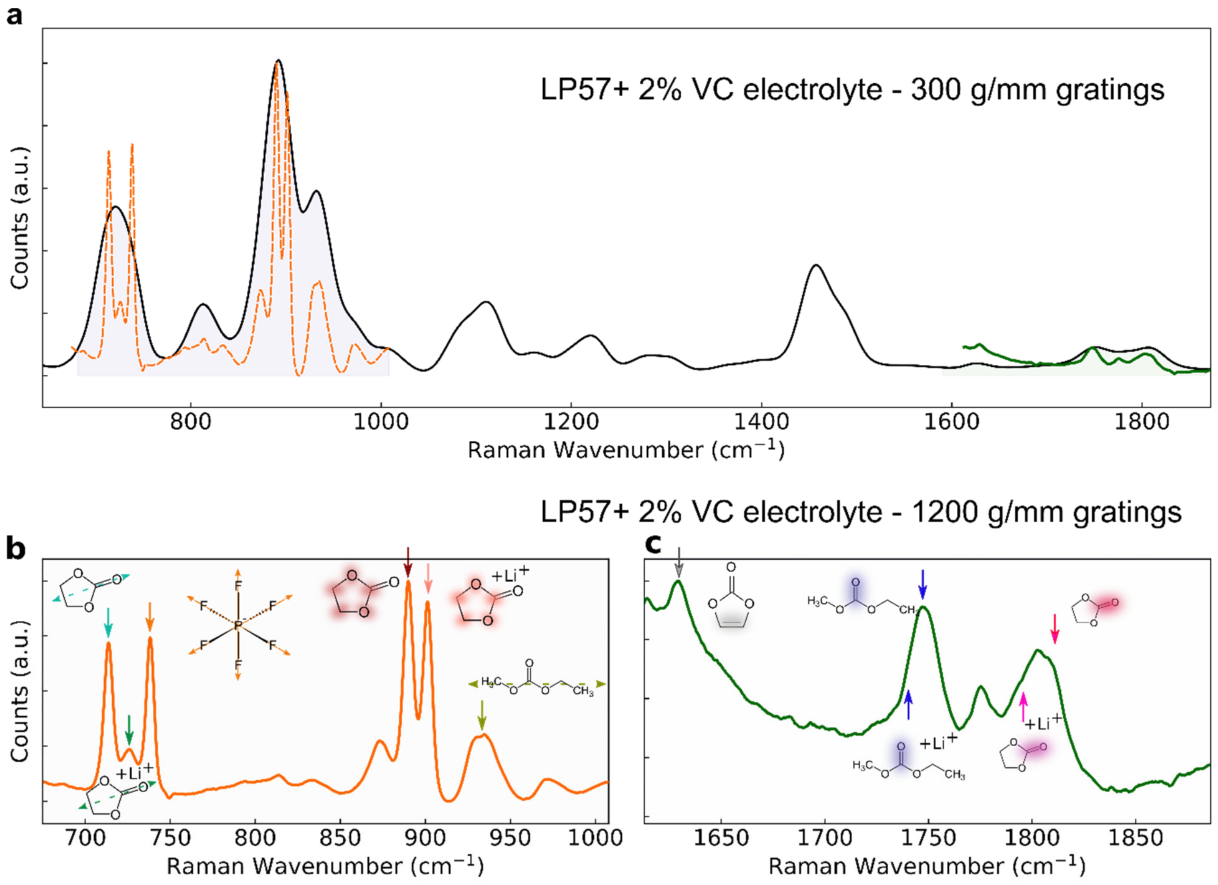

Supplementary Figure 6 | High-resolution Raman spectra. *Comparison between high- and low-resolution ex situ Raman spectra on non-cycled electrolyte, obtained using low (300 g/mm, 190 µm slit width, 27 cm*$^{-1}$ *resolution, black,* ***a****) and high (1200 g/mm, 90 µm slit width, 2.4 cm*$^{-1}$ *resolution, orange/green,* ***b*** *and* ***c****) resolution settings. Raman vibrations of interest for Li-ion solvation studies are highlighted in the high-resolution Raman scans (****b*** *and* ***c****). The high-resolution spectra in* ***b*** *and* ***c*** *have also been added as orange dashed and green lines in* ***a****. The integration time was 20 s for all spectra.*

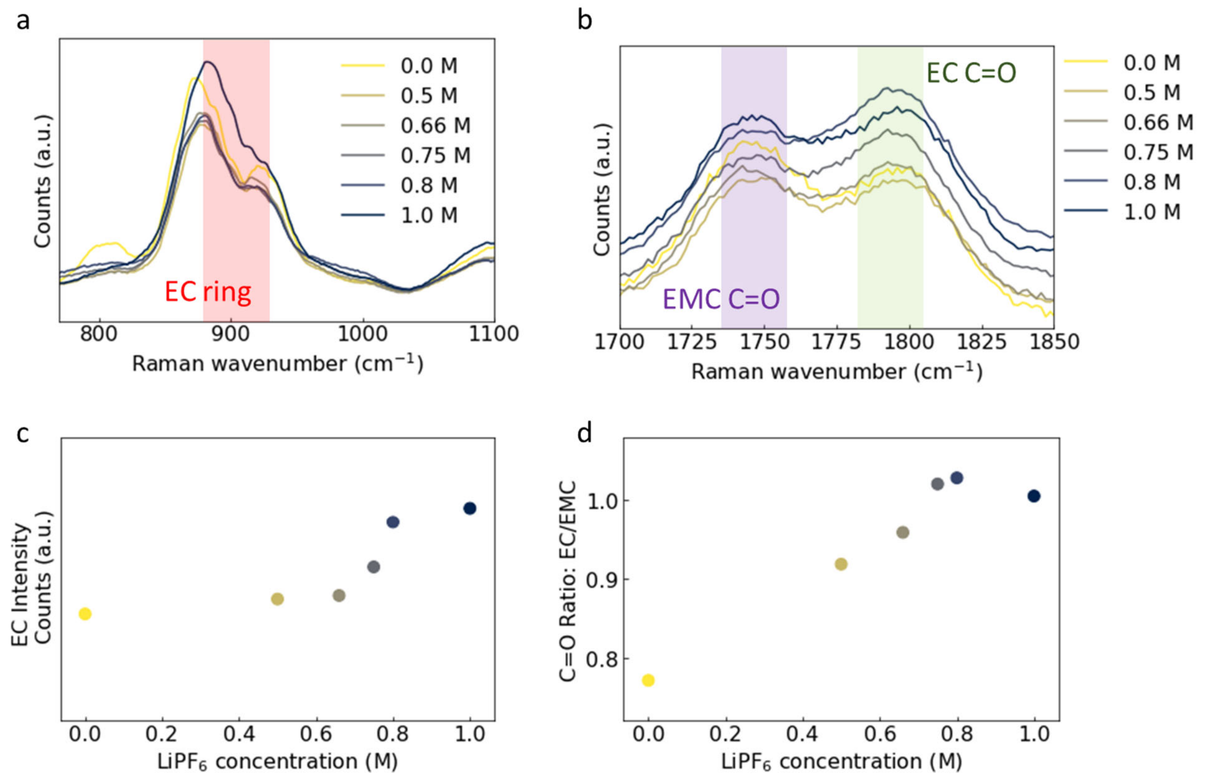

Supplementary Figure 7 | Ex situ calibration of Raman response. *The effect of Li concentration on the EC/EMC Raman spectra is studied by infiltrating prepared solutions of* $LiPF_6$ *salt in EC:EMC (3:7 (v)) in the HC-fibre sensor. (**a-b**) The EC breathing mode (shaded area in (**a**)) and the EMC- and EC C=O stretch modes (shaded areas in (**b**)) are measured for a range of [*$LiPF_6$*] concentrations between 0 and 1.0 M. The EC breathing mode (**c**) as well as the ratio between the EC and EMC (C=O) stretching modes (**d**) are observed to increase with [*$LiPF_6$*].*

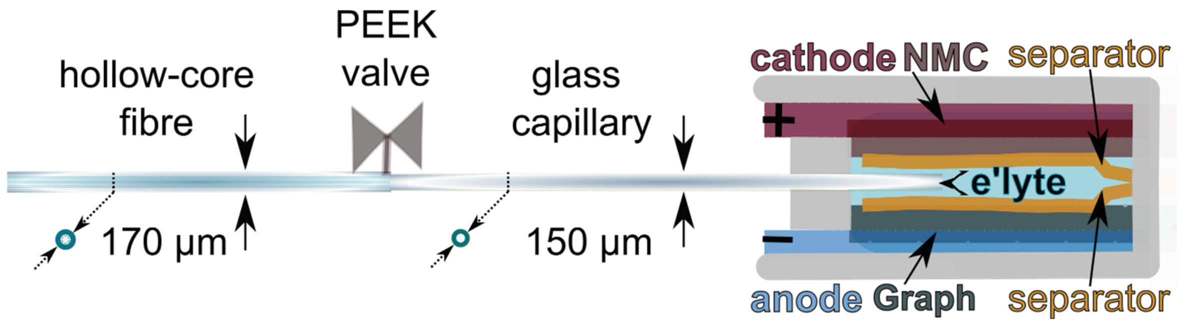

Supplementary Figure 8 | Alternative microfluidic configuration for a Li-ion cell. *The drawing depicts the stack layers arrangement and the position of the coated glass capillary used for experiments discussed in **Figure 6** in the main text. This configuration allows for separation between the hollow-core used for Raman detection and the capillary used for sampling the LP57 (VC-free) electrolyte.*

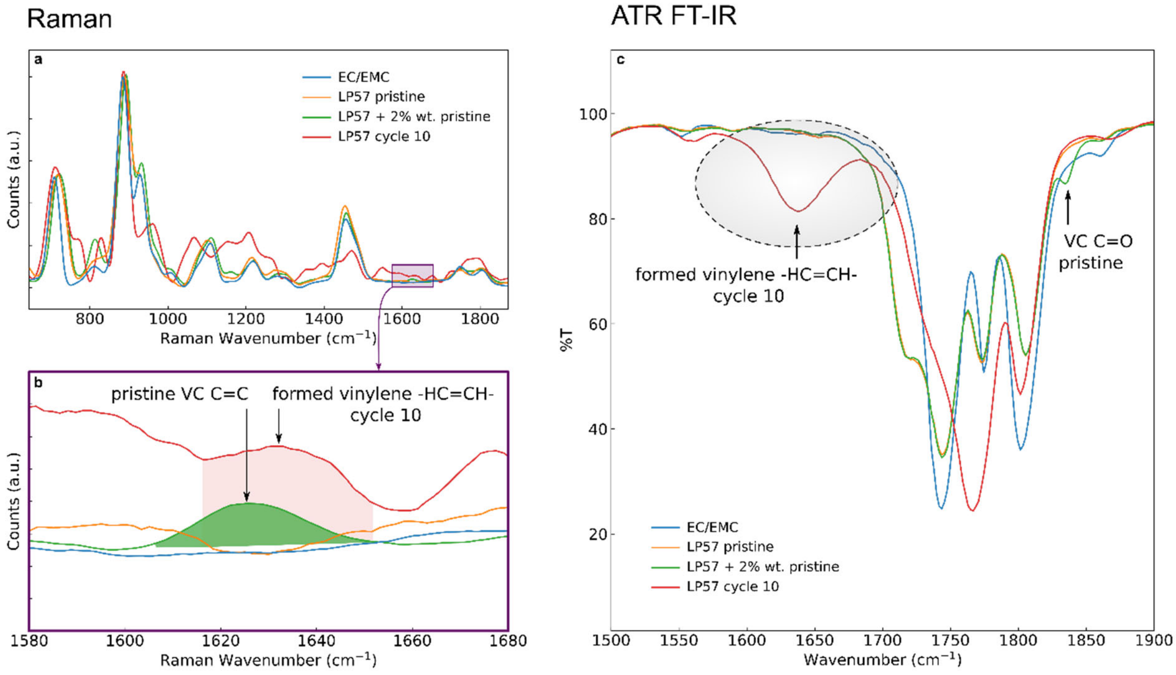

Supplementary Figure 9| Ex situ Raman and ATR FT-IR spectra. *Electrolyte samples were extracted from a coin cell (cell assembly, cycling parameters, and extraction procedure described in Methods section). Ex situ Raman spectra (**a**,**b**) were measured with the HC-fibre probe. ATR FT-IR spectra (**c**) were collected using a Shimadzu IRTracer-100 FT-IR spectrometer with QATR-10 ATR crystal.*

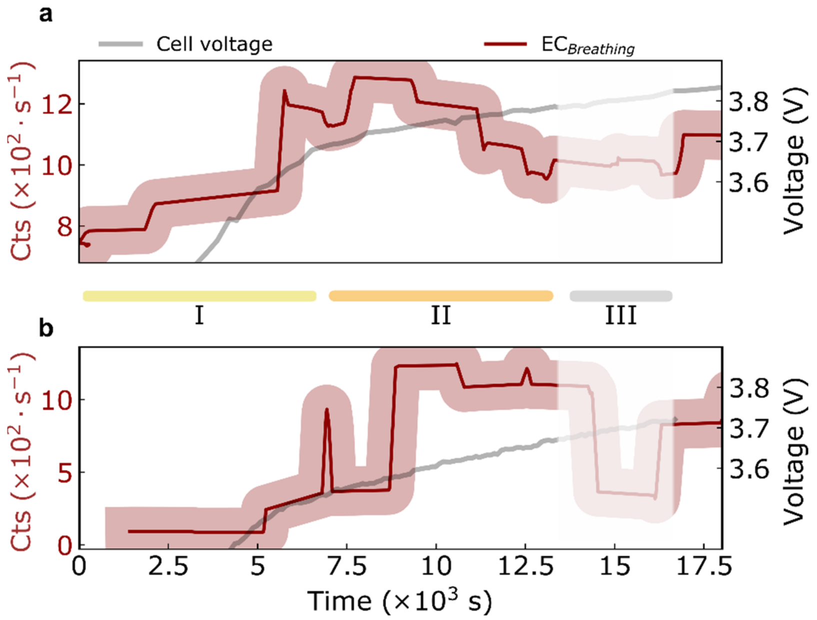

Supplementary Figure 10 | Representative measurements on different Li-ion pouch cells.
*Both experiments in (**a-b**) display a step increase in EC breathing mode intensity during charging (as in **Figure 3** in the main text), as well as a reduction in signal due to bubble formation at higher voltages (shaded areas). Sections* I-III *correspond to the same parts of the cycle as in **Figure 4** (main text).*

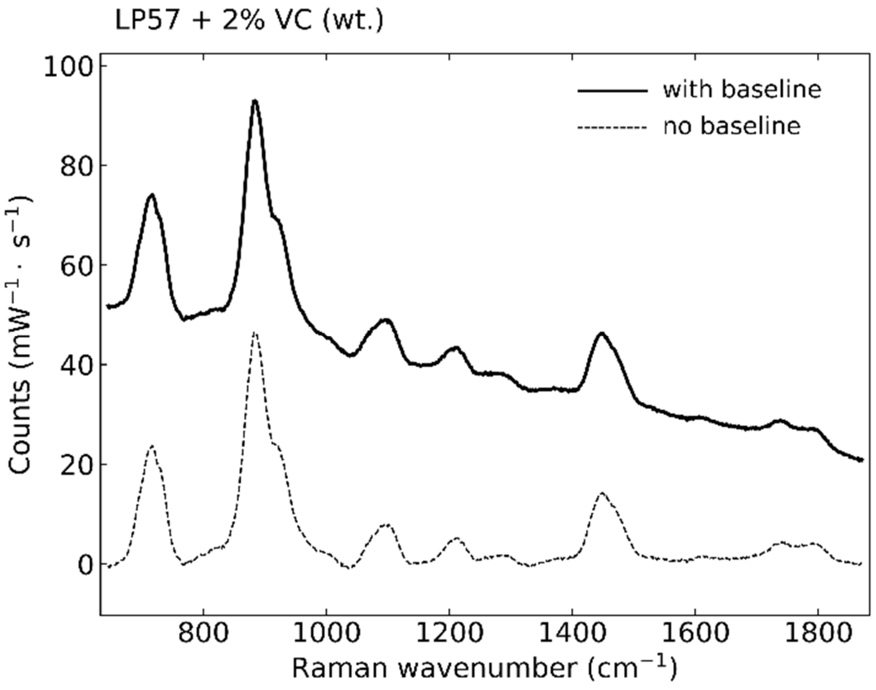

Supplementary Figure 11 | Raman spectra pre-processing and baseline subtraction. *Comparison between LP57 +VC 2% wt. Electrolyte before (continuous line) and after (dashed line) baseline subtraction. For each spectrum, the baseline is fitted with an order three polynomial curve. A Savitzky–Golay filter (window size 21 points, order 3) is finally applied to smooth the spectra. Counts are referenced to the input power of the pump laser.*

References

1. Litchinitser, N. M., Abeeluck, A. K., Headley, C. & Eggleton, B. J. Antiresonant reflecting photonic crystal optical waveguides. *Opt. Lett.* **27**, 1592–1594 (2002).
2. Thompson, L. M. *et al.* Quantifying Changes to the Electrolyte and Negative Electrode in Aged NMC532/Graphite Lithium-Ion Cells. *J. Electrochem. Soc.* **165**, A2732 (2018).
