# Supplementary material for: Hollow-core optical fibre sensors for operando Raman spectroscopy investigation of Li-ion battery liquid electrolytes
Source: Nat Commun. 2022 Mar 28;13:1651. doi: 10.1038/s41467-022-29330-4 (PMC8960792; doi:10.1038/s41467-022-29330-4)
Supplement: Supplementary file 3 — Description of additional Supplementary File [file 41467_2022_29330_MOESM3_ESM.pdf]

Descriptions of additional supplementary Information files

Supplementary Movie 1 | Gas bubble trapped in a hollow-core fibre. Video of side-scattered 785 nm laser light from a gas bubble trapped in a hollow-core fibre during electrolyte extraction. The movie has been acquired using a 12 MP chargecoupled device (CCD) sensor. (i.e. mobile phone camera).
